# Incidence of Iron Deficiency and the Role of Intravenous Iron Use in Perioperative Periods

**DOI:** 10.3390/medicina56100528

**Published:** 2020-10-12

**Authors:** Mirela Țigliș, Tiberiu Paul Neagu, Andrei Niculae, Ioan Lascăr, Ioana Marina Grințescu

**Affiliations:** 1 Department of Anaesthesiology and Intensive Care, Emergency Clinical Hospital of Bucharest, 014461 Bucharest, Romania; mirelatiglis@gmail.com (M.Ț.); ioana.grintescu@rospen.ro (I.M.G.); 2 Clinical Department No. 14, “Carol Davila” University of Medicine and Pharmacy, 050474 Bucharest, Romania; 3 Department of Plastic Surgery and Reconstructive Microsurgery, Emergency Clinical Hospital of Bucharest, 014461 Bucharest, Romania; ioan.lascar@gmail.com; 4 Clinical Department No. 11, “Carol Davila” University of Medicine and Pharmacy, 050474 Bucharest, Romania; 5 Department of Nephrology and Dialysis, “St. John” Emergency Clinical Hospital, 042122 Bucharest, Romania; niculaeandrei@yahoo.com; 6 Clinical Department No. 3, “Carol Davila” University of Medicine and Pharmacy, 050474 Bucharest, Romania

**Keywords:** iron deficiency, anemia, intravenous iron formulation, perioperative period

## Abstract

Iron deficiency is a major problem in worldwide populations, being more alarming in surgical patients. In the presence of absolute iron deficiency (depletion of body iron), functional iron deficiency (during intense bone marrow stimulation by endogenous or exogenous factors), or iron sequestration (acute or chronic inflammatory conditions), iron-restricted erythropoiesis can develop. This systemic review was conducted to draw attention to the delicate problem of perioperative anemia, and to provide solutions to optimize the management of anemic surgical patients. Systemic reviews and meta-analyses, clinical studies and trials, case reports and international guidelines were studied, from a database of 50 articles. Bone marrow biopsy, serum ferritin levels, transferrin saturation, the mean corpuscular volume, and mean corpuscular hemoglobin concentration were used in the diagnosis of iron deficiency. There are various intravenous iron formulations, with different pharmacological profiles used for restoring iron. In surgical patients, anemia is an independent risk factor for morbidity and mortality. Therefore, anemia correction should be rapid, with parenteral iron formulations—the oral ones—being inefficient. Various studies showed the safety and efficacy of parenteral iron formulations in correcting hemoglobin levels and decreasing the blood transfusion rate, the overall mortality, the postoperative infections incidence, hospitalization days, and the general costs.

## 1. Introduction

Iron deficiency (ID), a reduction of body iron levels, is a critical problem worldwide, affecting 4–30% of men, 10–43% of all women, and reaching 52% in pregnant women. Studies showed that iron deficiency complicates the management of almost one-third of surgical patients [1]. The prevalence of preoperative anemia varies from 26 to 75%, while after major surgery, it ascends to 90% [2]. It can occur due to excessive losses in patients with massive acute bleeding or chronic hemorrhages, malabsorption, insufficient intake in relation to increased needs, or functional deficiency due to a chronic disease (e.g., HIV, cancer). In surgical patients, the cause can be multifactorial [3]. Iron deficiency might or might not be associated with anemia (a decrease in hemoglobin levels and changes in erythrocytes morphology), often being unidentified or untreated. The first phase of iron deficiency does not manifest through anemia, which usually appears in a later stage [4]. Therefore, good perioperative patient management involves monitoring hemoglobin levels and iron status before surgery and, in case of major interventions, in the postoperative period [5].

In the perioperative period, anemia is an independent factor for morbidity and mortality. It is also related to an increased incidence of red blood cell transfusion, prolonged length of stay in hospital, and higher complications [6]. Fowler et al. published a meta-analyses on the influence of preoperative anemia on patients’ outcomes after major surgery, and concluded that it had a high incidence (about 39% of patients), being an independent risk factor for in-hospital mortality, acute kidney injury, and infections. In patients proposed for cardiac surgery, it was also an independent risk factor for stroke events [7].

In elective surgery, there might be enough time to correct anemia in the preoperative period, with oral and parenteral iron products or erythropoietic agents, but in major emergency surgery, there is no time for delays [6]. Anemia can also develop during hospitalization (major surgery, complications, blood sampling) and continues further after the patient’s discharge, if it is not properly corrected, leading to impaired functionality on the long-term. Therefore, in order to improve patient’s outcome, recent guidelines and programs promote the safety and efficacy of intravenous iron, in order to correct anemia, to reduce the need for blood transfusions, to decrease the rate of complications, and economically speaking, to reduce hospitalization costs [5,8].

## 2. Materials and Methods 

**Figure 1:**
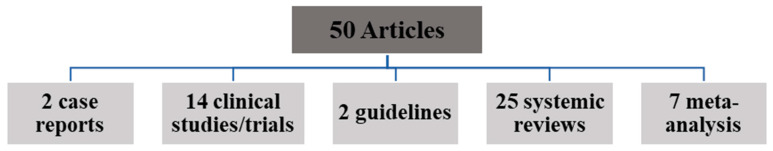
Articles used for analysis.

This systemic review was conducted to draw attention to the delicate problem of perioperative anemia, to highlight the risks it has on the patients’ evolution, to provide solutions for optimizing the clinical management of these surgical patients and to offer alternative solutions to blood transfusion. For this purpose, we used PUBMED database, searching for words and word combinations, in article titles or contents, like *“perioperative anemia”, “perioperative period”, “major surgery”, “iron deficiency”, “iron sequestration”, “intravenous iron”, “parenteral iron”, “iron molecule”, “iron formula”, “iron infusion”, “erythropoiectic agent”, “blood transfusion”, “hypersensitivity reactions”,* and *“erythropoiesis”*. In our review, we only studied English articles, using systemic reviews and meta-analysis, clinical studies and trials, case reports, and international guidelines. A total of 50 articles (Figure 1) were included and these were reviewed by three authors (M.Ț., T.P.N., and A.N.) and two other persons (I.L., I.M.G.) checked the eligibility. The FDA network was also consulted.

## 3. Results and Discussion

Parenteral iron formulations were first used in those with intolerance of, or unresponsiveness to oral iron. In the last century, intravenous iron showed its benefits in the perioperative period. It was used to rapidly correct the body iron levels and to improve hematopoiesis whenever important bleeding was anticipated or arose. Therefore, perioperative transfusion requirements and complication occurrences were reduced, improving the overall outcome of patients with chronic comorbidities [9,10].

In the following lines, important issues are presented—iron deficiency diagnosis, a short presentation of intravenous iron products, the role of parenteral iron use in the perioperative period, and the overall risks associated with intravenous iron products, in order to familiarize the clinician with the main aspects of surgical patients’ anemia, acquired iron deficiency, and the existent therapeutic alternatives. 

### 3.1. Iron Deficiency Diagnosis

Iron, a vital element, is involved in various essential biological processes of the body, like DNA synthesis, immune system functionality, erythropoiesis, hemoglobin synthesis, oxygen transport, energy metabolism, and the production of neurotransmitters [11]. Iron deficiency can commonly present as an absolute deficiency (when the iron stores are abolished) in the face of important blood loss. There can also be a functional or relative iron deficiency (increased erythropoietic response that exceeds the available iron supply) and an iron deficiency by sequestration (increased hepcidin levels, like in inflammatory diseases, induce iron retention in macrophages, or enterocytes) [12]. 

We should remember that iron exists in the body in many forms—circulating, stored intracellularly, and utilized, like part of the hemoglobin structure. The “gold standard” for iron deficiency diagnosis remains a bone marrow biopsy. It directly measures the iron stores that can be used in hematopoiesis, but it is a complicated, invasive, and low tolerated test that is used in rare cases. In clinical practice, the diagnosis relies mostly on serum biomarkers assessment (Table 1) [13].

One of the most sensitive tools for iron deficiency diagnosis is a serum ferritin level under 30 ng/mL, which is an expression of the depleted iron stores. Nevertheless, ferritin is also released when inflammation is present like in inflammatory bowel disease, autoimmune disease, or chronic renal failure, so normal, or elevated value does not always exclude iron deficiency [14,15]. Another important test is represented by transferrin saturation (TSAT), which measures the transported iron that is available for cell uptake but the reliability of TSAT in measuring iron status can also be reduced by a high inflammatory status [13]. Circulating iron, bound to its carrier (transferrin) can also be assessed, but its values can vary with the oral intake and the physiological necessities, and it can have a normal value even in the presence of depleted stores. Therefore, iron’s defining parameters should be drawn after an overnight fast [14]. C-reactive protein, used to assess the patient’s inflammatory status, can guide iron’s deficiency diagnosis. In the presence of low ferritin (30–100 ng/mL) and transferrin saturation levels (<20%), a level of C-reactive protein below 5 mg/L is a marker of absolute iron deficiency [16].

Others markers that can be used are a low value of the mean corpuscular volume (MCV) and mean corpuscular hemoglobin concentration (MCHC) (<280 g/L). The red cell distribution width (RDW) (variation of cells volume) is increased in initial phases and after the iron treatment initiation. The reticulocyte hemoglobin content (CHr), a reflexion of iron volume available for immediate erythropoiesis, is decreased (<28 pg) [17].

### 3.2. Intravenous Iron Products–Short Presentation

Regarding the chemical structure, intravenous iron formulas are colloidal suspensions with a core made of iron oxyhydroxide and a stabilizer, carbohydrate shell coating. The size of the core particle influences the iron lability, the smaller size being the most labile of bound iron [18]. The various intravenous iron formulations have a similar core but the chemistry, molecular weight of the carbohydrate coat, and the bonds with the core are different. After the intravenous administration, iron molecules dissociate from the carbohydrate shell coat and binds to specific proteins. According to various studies, iron–carbohydrate complexes are taken by macrophages of the reticuloendothelial system through endocytosis. In big steps, endosome fuses with lysosome, leading to iron cleavage from the complex. Then, the iron enters the cytoplasm of the macrophages and it is incorporated into ferritin or transported out and sequestrated into transferrin. After that, iron is transported into sites of usage [19]. The new iron formulas are more stable, strongly binding the iron molecule within the carbohydrate coat, and therefore, decreasing the iron release during infusion. It allows the infusion of larger doses in comparison to older formulas (Table 2) [18,20]. 

Ferric derisomaltose (Monoferric^®^), known since 2009 as iron isomaltoside 1000 (IIM)—Monofer^®^, was released under this name since January 2020. It is a “new” molecule with high stability, with a carbohydrate coat made of derisomaltose, with a molecular weight of 155 kDa and a plasma half-life of approximately 27 h. The maximum dose was 20 mg/kg for patients weighing <50% and 1000 mg in patients >50 kg, with a minimum time for administration of 20 min. It could replace the total required dose in one infusion or divided doses for correcting anemia, and does not require a test dose, according to the manufacturer [3,21,22,23].

Ferric carboxymaltose (FCM) (Ferinject^®^, Injectafer^®^) has a carboxymaltose shell coating, with a molecular weight of 150 kDa, a high stability, and a plasma half-life of approximately 8 h. The maximum single dose is 1000 mg, administered over 15 min. It also has the ability to replace the required iron dose in a single infusion [3,24].

Iron sucrose (IS) (Venofer^®^) is a medium stable molecule, with a sucrose shell coating, a molecular weight of 43 kDa, a plasma half-life of 5 h, a maximum dose of 200 mg with at least 15 min minimum time for the administration. This molecule has become available for use since 2000. It needs repeated infusions to ensure the required amount of iron (about 1 g) [3,22].

Low molecular weight iron dextran (LMW dextran) (Cosmofer^®^, InFed^®^) has a dextran coat, a high stability, and 400-kDa molecular weight with a plasma half-life of about 30 h. The maximum dose is 20 mg/kg, administered over a minimum of 4–6 h. It is available in the market since 1991 [18]. 

Ferumoxytol (Feraheme^®^, Rienso^®^) has a polyglucose sorbitol carboxymethyl ether shell coating, with a molecular weight of 750 kDa, a high stability, and a plasma half-life of 15 h. The maximum dose is 510 mg administered over a minimum of 15 min. This product is no longer available in the European Union [18].

Sodium ferric gluconate (SFG) (Ferrlecit^®^), being used since 1999, is a molecule with low stability, a gluconate-made coat, a 280-kDa molecular weight, and a plasma half-life of 1 h and a half. The maximum single dose is 125 mg, with a minimum administration time of 30–60 min. It does not replace the total necessary dose, so multiple repeated infusions are required [3,18]. 

There is no standardized recommendations for each formula—the selection depends on availability, the prescriber’s experience, the available time for correcting iron deficiency (elective or emergency surgery), the type of iron deficiency, and the patient allergenic profile [3,5,25,26,27,28].

### 3.3. The Role of Parenteral Iron Use in a Perioperative Period

As we previously emphasized, the perioperative period raised problems through the fact that there is no time to correct the iron deficiency with oral supplementation and many patients had zero response to these therapies due to the underlying comorbidities. Poor gastrointestinal tolerance led to the necessity of new intravenous product use in surgical patients, with a better tolerance profile, fewer adverse reactions, and rapid capacity of correcting iron deficiency [10,29,30].

A study published by Lee et al. has compared the efficacy and safety of ferric carboxymaltose (FCM) (500 mg in patients weighing <50 kg and 1000 mg in patients with >50 kg) versus iron sucrose (IS) (200 mg per session, maximum 600 mg per week), in treating preoperative anemia in gynecological patients. They concluded that 1000 mg dose of FCM leads to rapid correction of iron deficiency anemia, obtaining a hemoglobin level >10 g/dL in 7.7 days, compared to 10.5 days for IS. This allowed earlier surgical intervention, reduced the number of hospital visits (one visit for FCM, 3–8 visits for IS), and improved patient outcomes. Both formulations were safe, only being related to mild adverse events, like headaches. [31]. 

In cases of emergency surgery, with important bleeding or in cases requiring invasive procedures, it is recommended to restore body iron to improve postoperative recovery. High doses of parenteral iron are generally preferred (1000–1500 mg), allowing a rapid infusion (between 15 min and 1 h). For all nonselective surgical procedures, the therapy can be initiated or continued in the postoperative period [17]. Studies showed that for these cases, ferric carboxymaltose and iron isomaltoside 1000 are preferred over sodium ferric gluconate or iron sucrose. Intravenous iron administration hastened anemia correction, better replenished the iron stores, and reduced adverse event appearance, as compared to oral products [28].

In another multicenter study (IVICA trial), Keeler et al. analyzed the role of preoperative iron administration in improving the quality of life for patients after colorectal cancer surgery. Authors pointed out that the intravenous route (55 patients) is more efficient than oral iron (61 patients) in correcting hemoglobin level and improving patients’ outcomes. Using the parenteral formulas, they observed rapid improvement in clinical quality of life scores on short and long-term evolution, possibly in relation to anemia correction. Intravenous iron increased the hemoglobin levels more rapidly than oral formulas, permitting an earlier surgical intervention or adjuvant therapies [32]. 

Intravenous iron agents have showed their usefulness in orthopedic surgery, and have become “a state of the art”, as described by Muñoz et al. These are used on a daily basis in patients at risk for perioperative anemia, decreasing the need for blood transfusion and the number of transfused units, by rapidly correction the hemoglobin concentration. The postoperative recovery is hastened, the length of stay is reduced, and the cost-effectiveness is significant [33,34,35].

A recent meta-analysis published by Schack et al., in which 5413 studies were screened, examined the role of perioperative parenteral iron therapy in cases of acute non-cardiac surgery. A total of 3044 surgical patients (especially orthopedic interventions) were enrolled in these studies. A decrease in 30-days mortality, allogenic blood transfusion, and a lower rate of postoperative infections were observed. After the analysis, no statistical difference was observed with regards to the postoperative hemoglobin level or the hospital’s length of stay [36].

### 3.4. The Overall Risks Associated with Intravenous Iron Products

During iron infusion, there is a risk of hypersensitivity reaction appearance or iron overload [37,38,39]. Studies showed that the incidence of adverse reactions is 1 in 200,000 patients, with a prevalence of <0.1% [24,40,41,42]. The infusion rate plays a key role. Based on experts’ consensus, an infusion time extent from 15 min to one hour is recommended for the first dose. Then, the infusion time stated in the drug monograph should be respected [25].

A recent analysis, published by Achebe and DeLounghery, studied the risk of severe hypersensitivity reaction appearance related to intravenous iron used in 5247 patients. The authors concluded that there were no statistical differences regarding the severity or risk of adverse reaction appearance between various types of iron formulations [43]. Girreli et al. pointed out that if we compared the frequency and gravity of adverse events related to intravenous iron administration and red blood cell transfusion, we would observe that the rate of a major complication was lower in the iron group [3].

Mild reactions are characterized by flushing, urticaria and itching, joint pain, and chest tightness, and disappear if the infusion is stopped or the rate is lowered (Table 3) [10,44]. Cases with moderate reactions require stopping the infusion, and, in the face of marked hypotension, tachycardia, dyspnea, cough, and important chest tightness, intravenous fluids and steroids might be required [45]. Rampton et al. published a review in which they highlighted that life-threatening manifestations (cardiac arrest, wheezing, coma), which needed advanced cardiac life support, were extremely rare [46]. Delayed reactions (after 30 min since the treatment was finished) were also extremely rare. They were unspecific and manifest through fever, headache, myalgia, or arthralgia [26,47,48].

History of previous hypersensitivity reactions, atopy, and mastocytosis were cited as risk factors. There were also some elements related to increased severity of the adverse events—male sex, the concomitant use of beta-blockers or ACE inhibitors, older age and psychological liability, patients with behavioral conditions that are often non-compliant with the treatment [11,49].

A recent randomized trial, published by Wolf et al. showed that ferric carboxymaltose had a particular side effect, hypophosphatemia, being related to the stimulation of fibroblast growth factor 23 [11]. It is often asymptomatic, but in rare cases, it can lead to profound fatigue, muscle weakness, bone fractures, and osteomalacia [50,51].

## 4. Conclusions

The intravenous iron formulation are safe for use in the perioperative period. The use of one formula to the detriment of others is not standardized yet, the selection criteria especially being the patient profile, the prescriber’s experience, the drug availability, and the time left until the surgery. The new drugs are available in a single, higher dose that allow rapid correction of anemia, granting early surgery, and reducing the number of hospital visits. The correction of iron deficiency in surgical patients is vital for overall outcome, being related with a reduced need of allogeneic blood transfusion and all associated complications, faster recovery after surgery, low rate of infections, reduced length of stay in the hospital, reduced rate of complications, and a lower cost. 

## Figures and Tables

**Table 1:** Serum biomarkers used to diagnose iron deficiency.

“Gold Standard” Method	Usual Biomarkers	Other Biomarkers
Bone marrow biopsy	Serum ferritin	Mean corpuscular volume (MCV)
	Transferrin saturation (TSAT)	Mean corpuscular haemoglobin concentration (MCHC)
	Serum iron	Red cell distribution width (RDW)
	C-reactive protein	Reticulocyte haemoglobin content (CHr)
		Zinc protoporphyrins (ZPP) in the red cell

**Table 2:** Intravenous iron formulas, usual doses, and time of administration.

Intravenous Iron Formula	Dosage and Minimum Administration Time
1. Ferric carboxymaltose (Ferinject^®^, Injectafer^®^)	1000 mg in 15 min
2. Ferric derisomaltose (Monoferric^®^)	1000 mg in at least 20 min (in patients > 50 kg) 20 mg/kg in at least 20 min (in patients < 50 kg)
3. Iron sucrose (Venofer^®^)	200 mg in 30 min
4. Low molecular weight iron dextran (LMW dextran) (Cosmofer^®^, InFed^®^)	20 mg/kg in 4–6 h
5. Sodium feeric gluconate (Ferrlecit^®^)	125 mg in 30–60 min
6. Ferumoxytol (Feraheme^®^, Rienso^®^)	No longer in use

**Table 3:** Frequent adverse reactions related to parenteral iron administration.

Adverse Reactions	Usual Treatment
1. flushing	➢ lowering the infusion rate ➢ stopping the infusion
2. urticaria	
3. itching	
4. joint pain	
5. chest tightness	
